# Neonatal survival and mortality predictors among low-birth-weight neonates: A Retrospective cohort study from Suhul Hospital, northern Ethiopia

**DOI:** 10.1371/journal.pone.0354163

**Published:** 2026-08-03

**Authors:** Abrahaley Brhane Gebrekidan, Negasi Asres Mesfin, Kiros Gereziher Arafayne, Getachew Mebrahtu Welay

**Affiliations:** 1 Department of Human Nutrition, School of Public Health, College of Health Sciences, Aksum University, Aksum, Ethiopia; 2 Department of Epidemiology and Biostatistics, School of Public Health, College of Health Sciences, Aksum University, Aksum, Ethiopia; Ibaraki Children’s Hospital: Ibaraki Kenritsu Kodomo Byoin, JAPAN

## Abstract

**Background:**

Neonates, defined as infants within the first 28 days of life, are especially vulnerable in conflict-affected settings. Globally, about 2.3 million neonatal deaths occur annually, with nearly 98% occurring in low- and middle-income countries. Despite several studies in this area, context-specific evidence remains limited, and knowledge and data gaps continue to hinder efforts to address health inequalities in resource-limited settings. This study, therefore, aims to evaluate neonatal mortality and identify its key predictors.

**Objective:**

This study aimed to assess neonatal mortality and identify its associated risk factors.

**Materials and methods:**

A hospital-based retrospective cohort study was conducted among 329 neonates admitted between 2021 and 2023. Data were extracted from medical charts, registers, and maternal and neonatal history sheets. Kaplan-Meier survival curves and Cox regression models were used for analysis. Log-rank test and proportional hazards assumption were evaluated. Predictors of neonatal mortality were identified using Cox proportional hazards regression model, with statistical significance set at p < 0.05.

**Results:**

The overall neonatal mortality rate was 216 per 1,000 live births (95% CI: 190.5–241.5), with a median survival time of 52 days. Multivariable Cox regression identified respiratory distress syndrome (RDS) and delayed initiation of breastfeeding as significant predictors of neonatal mortality. RDS was associated with an increased risk of death [AHR = 2.00, 95% CI: 1.16–3.46]. Compared with breastfeeding initiated within one hour of birth, delayed initiation of breastfeeding was associated with a higher risk of mortality [AHR = 6.23, 95% CI: 3.03–12.82], whereas formula feeding showed a similar trend that did not reach statistical significance [AHR = 2.602, 95% CI: 0.835–8.10].

**Conclusion and recommendations:**

Neonatal mortality in this study was substantially high. Delayed initiation of breastfeeding and respiratory distress syndrome were identified as significant predictors of neonatal death. Public health interventions should focus on addressing modifiable risk factors for neonatal mortality. Strengthening postnatal maternal health education, promoting early initiation of breastfeeding, and increasing community awareness are essential. In addition, prioritizing respiratory distress syndrome management through effective infection prevention and control measures, quality neonatal care, and timely access to oxygen and surfactant therapy could enhance neonatal survival.

## Introduction

Neonates, defined as infants from birth through 28 days of age [1], are highly vulnerable to risks stemming from conflict-related maternal and neonatal exposures. Newborns exhibit distinct immunological and physiological characteristics compared to adults, rendering them particularly vulnerable to diseases and death. These inherent vulnerabilities contribute to a global neonatal health crisis, with an estimated 2.3 million deaths occurring annually and approximately 6,300 deaths each day [1]. The burden is disproportionately concentrated in low- and middle-income countries (LMICs), accounting for nearly 98% of neonatal deaths, where limited access to quality healthcare for preventable conditions is further compounded by conflict and displacement [2–4]. In LMICs, including Ethiopia, the neonatal mortality rate is estimated at 27 deaths per 1,000 live births, with nearly 75% of all neonatal deaths occurring within the first week of life [5,6].

Shire-Inda Selassie, located in Northwest Tigray, Ethiopia, has been affected by armed conflict for several years. The area faces a shortage of specialized referral and university teaching hospitals, weak health system governance, and limited availability of essential neonatal care equipment, including oxygen plants, incubators, ventilators, and other critical medical devices. These systemic challenges, exacerbated by the ongoing conflict, have significantly compromised the quality and accessibility of neonatal healthcare, thereby increasing the risk of neonatal mortality [7].

Neonatal deaths among low-birth-weight (LBW) infants arise from conflict-related, neonatal, and maternal factors. Conflict-related factors contribute significantly to neonatal mortality, particularly among low-birth-weight infants [6,8], by disrupting health systems and limiting access to essential neonatal care resources, such as incubators, reliable electricity, and supply chains for essential medicines and vaccines [9,10]. The absence of incubators, often due to looting or power outages, directly affects ambient temperature regulation, leading to hypothermia or hyperthermia and subsequent neonatal death [7,11]. Similarly, shortages of essential medicines and vaccines predispose neonates to infections, including sepsis and vaccine-preventable diseases, further increasing mortality. Healthcare workers, including skilled midwives and birth attendants, frequently flee conflict zones to seek safety and employment elsewhere, leaving health facilities understaffed and contributing to higher neonatal mortality rates [12,13].

Specific neonatal complications are further exacerbated in resource-limited and conflict-affected settings. LBW is strongly associated with neonatal mortality because of its susceptibility to life-threatening complications. Birth asphyxia is a major contributor to neonatal mortality, particularly in settings where access to skilled birth attendants and timely neonatal resuscitation is limited. Respiratory distress syndrome is also a common cause, particularly in conflict-affected settings and displaced and overcrowded populations, where access to oxygen, ventilator support, surfactant therapy, and other essential neonatal care services is limited. Evidence suggests that male neonates are at a higher risk of death than female neonates, while complications such as asphyxia and pneumonia further increase the risk of mortality [14].

Maternal factors play a decisive role in neonatal outcomes in unstable situations, often by limiting access to antenatal care and worsening maternal malnutrition due to food insecurity. Pregnant women face substantial barriers to accessing antenatal services, including restricted transportation and insecurity-related challenges. Additionally, chronic maternal illnesses, such as malaria and HIV, increase the risk of low birth weight, which subsequently predisposes neonates to adverse birth outcomes and poor survival. The interdependence between maternal and neonatal health underscores their inseparable relationship in determining neonatal survival [15].

Efforts to reduce neonatal mortality have been implemented both globally and locally. Ethiopia has made notable progress through the adoption of community-based health extension programs and the promotion of institutional delivery strategies, contributing to a reduction in neonatal mortality from 48 to 27 per 1,000 live births between 2000 and 2020 [16].

Despite several studies and interventions addressing neonatal mortality, context-specific evidence gaps and data limitations remain major challenges in reducing health inequalities in low-resource, conflict-affected contexts. Moreover, existing predictive models of neonatal mortality often assume stable health systems, which may not be applicable to fragile and conflict-affected settings. Therefore, this study aimed to assess neonatal mortality and identify its key predictors.

## Materials and methods

### Study setting, design, and population

This hospital-based retrospective cohort study was conducted at Suhul Hospital in Shire-Inda Selassie, located in the conflict-affected northwest Tigray region of Ethiopia. The hospital serves over eight districts within its catchment area. Before the onset of the conflict, the Neonatal Intensive Care Unit (NICU) recorded an average annual admission rate of 1,244 neonates. According to the Obstetrics and Gynecology Department, facility-based delivery coverage was approximately 80%. To ensure data consistency and standardization, records were obtained from a single healthcare facility.

### Eligibility criteria

The study included all newborns weighing less than 2,500 grams who were admitted to Suhul Hospital between 2021 and 2023, a period that largely coincided with the conflict. Only records with complete medical documentation and deliveries occurring within this timeframe were included.

Records were excluded if the birth weight was 2,500 grams or more. Additionally, medical records with incomplete or missing values were excluded from the study. Neonates whose admission or discharge dates were unknown were not considered for analysis. Neonates with congenital anomalies that could affect survival and those born at or before 22 weeks of gestation were also excluded.

### Sampling and sample size determination

Given the retrospective study design, a total population sampling technique was employed. All eligible records of low-birth-weight neonates admitted between 2021 and 2023 were included in the study. To ensure robust statistical power, all eligible records were utilized. The final total sample comprised 329 neonates with complete medical records.

### Data sources and data collection tools

Data were collected using a structured checklist developed for the study. Information was extracted from hospital records, including neonatal and maternal medical charts, vital sign records, history sheets, and NICU and maternity medical registration books.

The extracted data were entered into the KoboToolBox application for data collection and management. The completed dataset was then downloaded from KoboCollect into Microsoft Excel and subsequently imported into SPSS version 27 and Stata version 15 for data cleaning, management, and final analysis.

### Ascertainment of neonatal death

The time-to-event was defined as the period from admission to discharge. Follow-up time, measured in days, extended from the date of admission until the occurrence of the event of interest (death). Death was coded as 1, while censored outcomes, including neonates with undetermined status, defaulters, survivors, and transfers, were merged and coded as 0. The merging of multiple classifications was performed to avoid wider confidence intervals.

Death was confirmed according to the Suhul Hospital’s standard guidelines. The cause of death was determined and documented by pediatricians or neonatal nurses on duty. Once a neonatal death was confirmed, it was documented in both the admission log and the NICU record. Data collectors subsequently gathered the raw data from these records.

### Measurement of exposure variables

Neonatal and maternal exposure factors were obtained from maternal and neonatal history sheets, medical charts, and registration books.

Demographic factors: Neonatal sex, residence (urban/rural), maternal age, neonatal age, and distance from the referring health facility or the district of residence to the respective hospital were extracted from the hospital records.

Obstetric history factors: Antenatal care (ANC) follow-up, place of delivery, mode of delivery, number of pregnancies or gravidity, history of abortion, and delivery complications were extracted directly from respective maternal and neonatal records.

Neonatal clinical factors: Gestational age, birth weight (<1500 g and 1500–2499 g), 5-minute APGAR score, presence of sepsis, birth asphyxia, prematurity, respiratory distress syndrome, hypothermia, initiation of breastfeeding, and weight at discharge were extracted from the medical records and NICU registers. Clinical diagnoses were based on the standard treatment guidelines followed by the hospital, which are aligned with the WHO recommendations.

Neonatal weight was measured using a calibrated digital infant scale with a sensitivity of 1 g. Socioeconomic status and maternal HIV status were not included because of the lack of documentation in the secondary data sources.

### Data quality assurance and statistical analysis

All records were manually reviewed for completeness and consistency of the data. Data cleaning was performed to check missing, inconsistent, or implausible values. Statistical analyses were conducted using SPSS version 27 and Stata version 15.

Descriptive statistics were used to summarize the study variables. Continuous variables were described using the mean, median, standard deviation, and life tables. Categorical variables were analyzed using frequencies, proportions, and percentages. Kaplan-Meier survival curves and the log-rank test were used to estimate survival probabilities and to determine if any difference exists between groups.

The Cox proportional hazards regression model was used to identify the predictors of mortality. Univariate Cox regression analysis was performed prior to multivariable modeling. Proportional hazards assumption was tested using Schoenfeld residuals (p > 0.05) and visual inspection of log-log survival plots. No significant time-dependent covariates were identified (p > 0.05). Variables with p-values < 0.05 in the final multivariable model were considered statistically significant predictors of neonatal death. Despite implementing data quality assurance and appropriate data analysis techniques, the recent armed conflict in the Tigray region adversely affected both data quality and service delivery at the hospital. Frequent power interruptions, supply shortages, and the loss of key neonatal equipment disrupted routine care and documentation processes. Reduced staffing due to displacement further strained the health system, contributing to inconsistent or delayed records.

### Ethical considerations

Ethical approval was obtained from the Institutional Review Committee (IRC) of Aksum University, College of Health Sciences, School of Public Health (Ref. No. IRC 012/2023). As the study was based on retrospective record review and involved no direct patient contact, the IRC waived the requirement for written or verbal informed consent. To ensure confidentiality, all personal identifiers were removed from the dataset before analysis. The data were anonymized and stored securely in password-protected files accessible only to the research data analyst team.

## Results

### Assessment of neonatal mortality among low-birth-weight neonates

This study was conducted in a conflict-affected, resource-limited setting where access to public healthcare is severely constrained by recurring political instability. Of the 346 neonatal records reviewed, 329 (95%) met the inclusion criteria and were included in the final analysis. As shown in Table 1, most mothers (218, 66.3%) resided in rural areas. Among male low-birth-weight neonates (n = 188), 46 (24.5%) died, accounting for 64.8% of all neonatal deaths.

**Table 1:** Descriptive analysis of neonatal, maternal, and medical care practice factors associated with mortality among low-birth-weight neonates admitted to Suhul Hospital, Shire-Inda Selassie, Northern Ethiopia, 2023 [N = 329].

Characteristics	Variable	Category	Number (%)	Status of LBW neonates n (%)	
				Censored	Died
Neonatal characteristics	Maternal residence	Rural	218 (66.3%)	175 (80.3%)	43 (19.7%)
		Urban	111 (33.7%)	83 (74.8%)	28 (25.2%)
	Sex of neonate	Female	141 (42.9%)	116 (82.3%)	25 (17.7%)
		Male	188 (57.1%)	142 (75.5%)	46 (24.5%)
	Weight at birth	1500-2499	298 (90.6%)	243 (81.5%)	55 (18.5%)
		<1500	31 (9.4%)	15 (48.4%)	16 (51.6%)
	Prematurity	No	119 (36.2%)	95 (79.8%)	24 (20.2%)
		Yes	210 (63.8%)	163 (77.6%)	47 (22.4%)
	Gestational age	≥37 weeks	59 (17.9%)	50 (84.7%)	9 (15.3%)
		34-36	142 (43.2%)	117 (82.4%)	25 (17.6%)
		<34	128 (38.9%)	91 (71.1%)	37 (28.9%)
	5-minute APGAR score	≥7	192 (58.4%)	166 (86.5%)	26 (13.5%)
		<7	137 (41.6%)	92 (67.2%)	45 (32.8%)
Medical conditions and care practice characteristics	Birth asphyxia	No	278 (84.5%)	235 (84.5%)	43 (15.5%)
		Yes	51 (15.5%)	23 (45.1%)	28 (54.9%)
	Respiratory distress syndrome (RDS)	No	285 (86.6%)	241 (84.6%)	44 (15.4%)
		Yes	44 (13.4%)	17 (38.6%)	27 (61.4%)
	Sepsis (Infection)	No	254 (77.2%)	195 (76.8%)	59 (23.2%)
		Yes	75 (22.8%)	63 (84%)	12 (16%)
	Hypothermia	No	153 (46.5%)	124 (81%)	29 (19%)
		Yes	176 (53.3%)	134 (76.1%)	42 (23.9%)
	Care provided	>5	60 (18.2%)	54 (90%)	6 (10%)
		3-5	198 (60.2%)	160 (80.8%)	38 (19.2%)
		<3	71 (21.6%)	44 (62%)	27 (38%)
	Initiation of breast-feeding	Within 1 hour	120 (36.5%)	108 (90%)	12 (10%)
		After 1 hour	137 (41.6%)	124 (90.5%)	13 (9.5%)
		Formula feeding	13 (4%)	9 (69.2%)	4 (30.8%)
		Delayed IBF	59 (17.9%)	17 (28.8%)	42 (71.8%)
	Discharge weight	≥2500	30 (9.1%)	28 (93.3%)	2 (6.7%)
		1500-2499	269 (81.8%)	222 (82.5%)	47 (17.5%)
		<1500	30 (9.1%)	8 (26.7%)	22 (73.3%)

The overall neonatal mortality rate among low-birth-weight neonates was 216 deaths per 1,000 live births during hospitalization (95% CI: 190.5–241.5), with male neonates accounting for 139.8 deaths per 1,000 live births. Of the total cohort, 258 neonates (78.4%) were censored, including 247 (75.1%) discharged following recovery, 5 (1.5%) referred for advanced care, and 6 (1.8%) discharged against medical advice.

**Fig 1:**
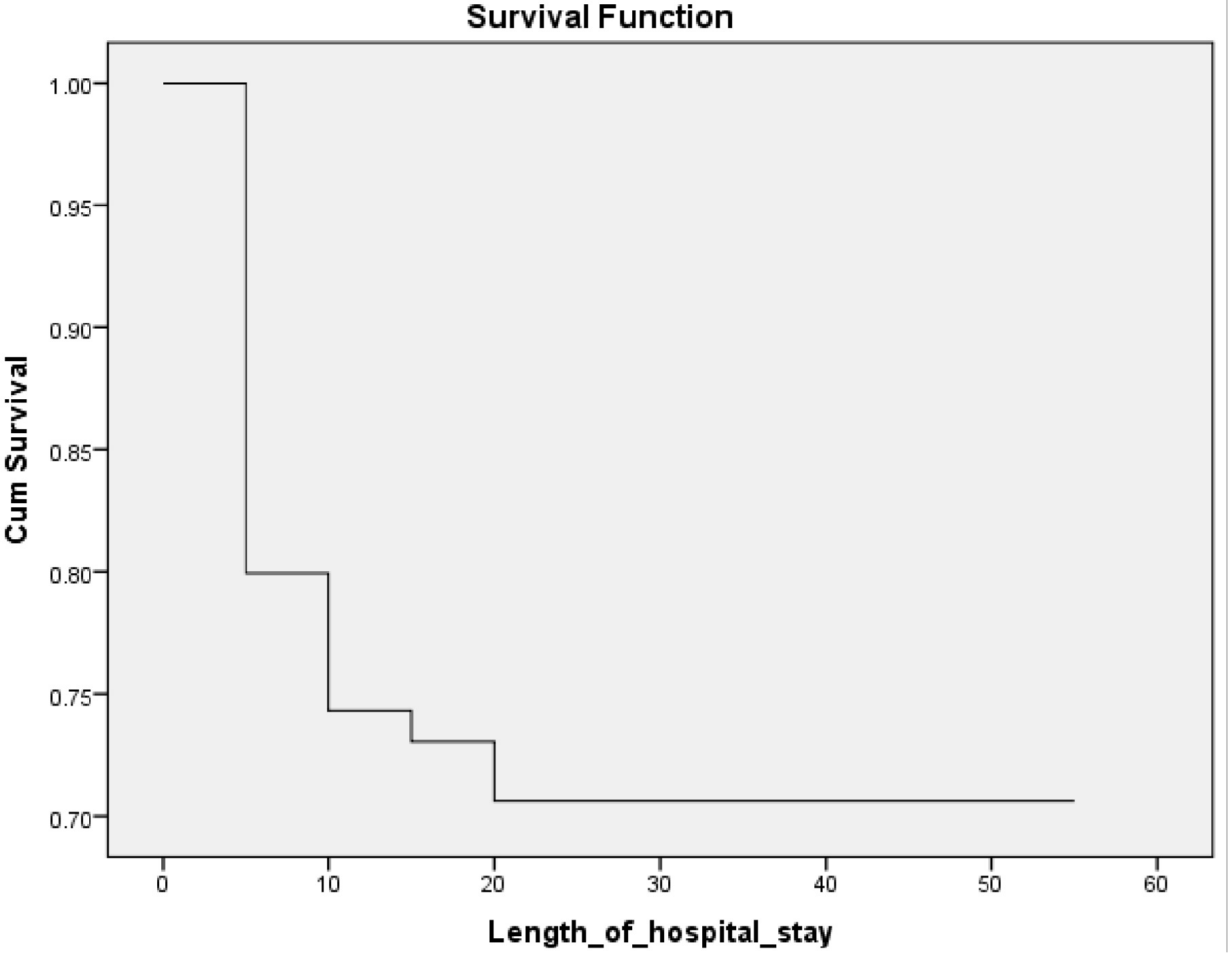
Overall Kaplan-Meier survival curve depicting the survival probability of low-birth-weightneonates admitted to Suhul Hospital, Shire-IndaSelassie, Northern Ethiopia, 2023.

The 329 neonates contributed a total of 2,346 neonate-days of observation, yielding an incidence rate of 30.26 deaths per 1,000 person-days (95% CI: 20.39–38.19). Among the 71 recorded deaths, 38 (53.5%) occurred within the first 24 hours of life, 20 (28.2%) occurred between the second and third days, and 11 (15.5%) occurred between the fourth and seventh days. Kaplan–Meier survival analysis indicated that the probability of survival was lowest on the first day of admission, with a median survival time of 52 days. Cumulative survival probability declined progressively over time, with rates of 88% on day 1 (95% CI: 0.84–0.91), 84% on day 2 (SE: 0.0199; 95% CI: 0.80–0.88), 81% on day 3 (95% CI: 0.77–0.85), and 72.96% by day 18 (95% CI: 0.65–0.79) (see Fig 1).

### Kaplan–Meier survival analysis stratified by key clinical risk factors

**Fig 2:**
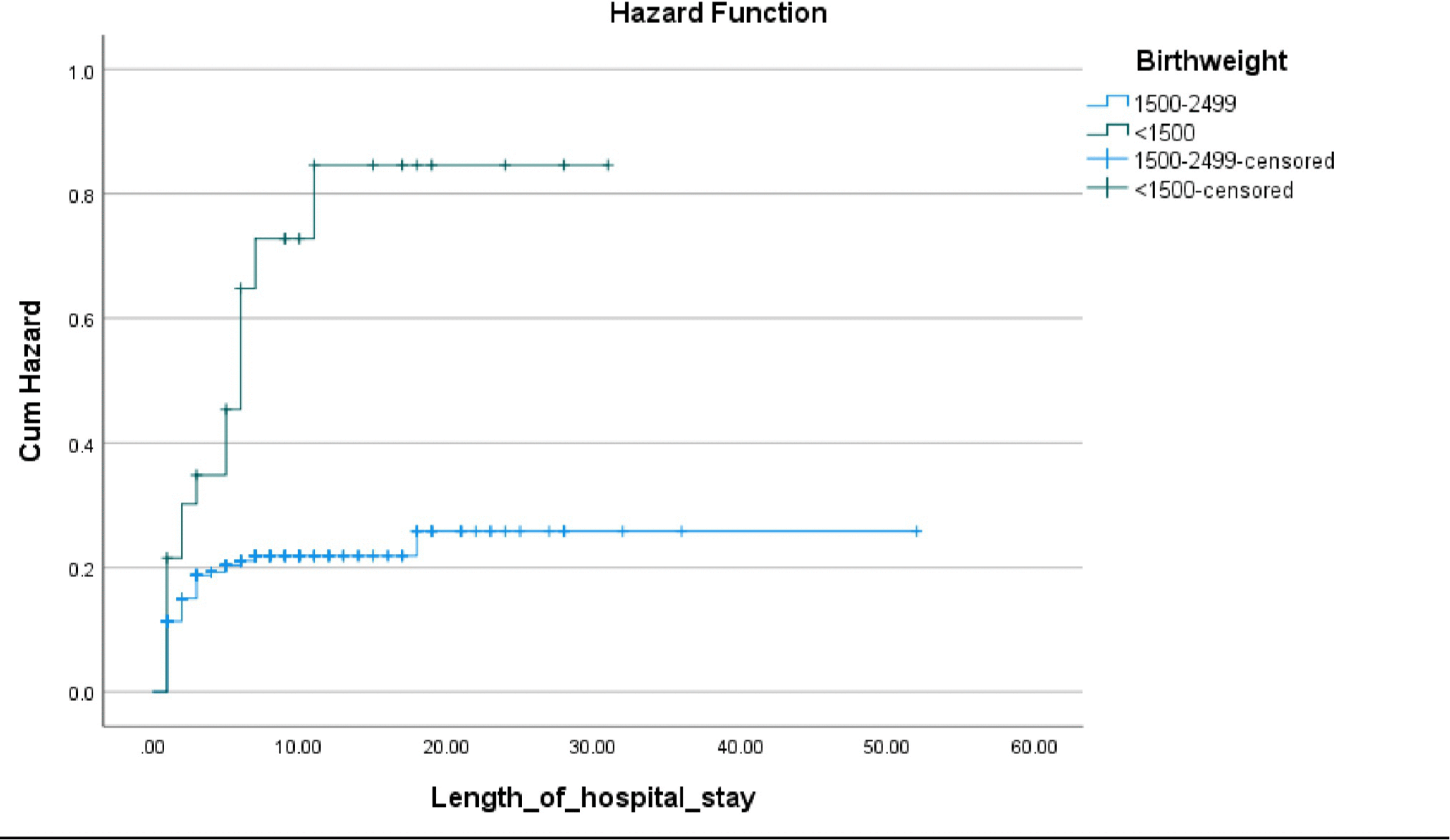
Kaplan-Meier hazard curves comparing time-to-event outcomes stratified by birth weight category among low-birth-weight neonates admitted to Suhul Hospital, Shire-Inda Selassie, Northern Ethiopia, 2023.

The log-rank test demonstrated significant differences in survival distributions across exposure variables among low-birth-weight neonates. As illustrated by the KM hazard curve (see Fig 2), marked differences in survival were observed across birth weight categories. Neonates weighing <1500 g had a median survival time of 15.6 days (95% CI: 10.5–20.7), whereas those weighing 1500–2499 g had a substantially longer median survival time of 41 days (95% CI: 38–44). Overall, neonates with birth weights <1500 g exhibited a notably higher risk of mortality compared with those weighing 1500–2499 g.

**Fig 3:**
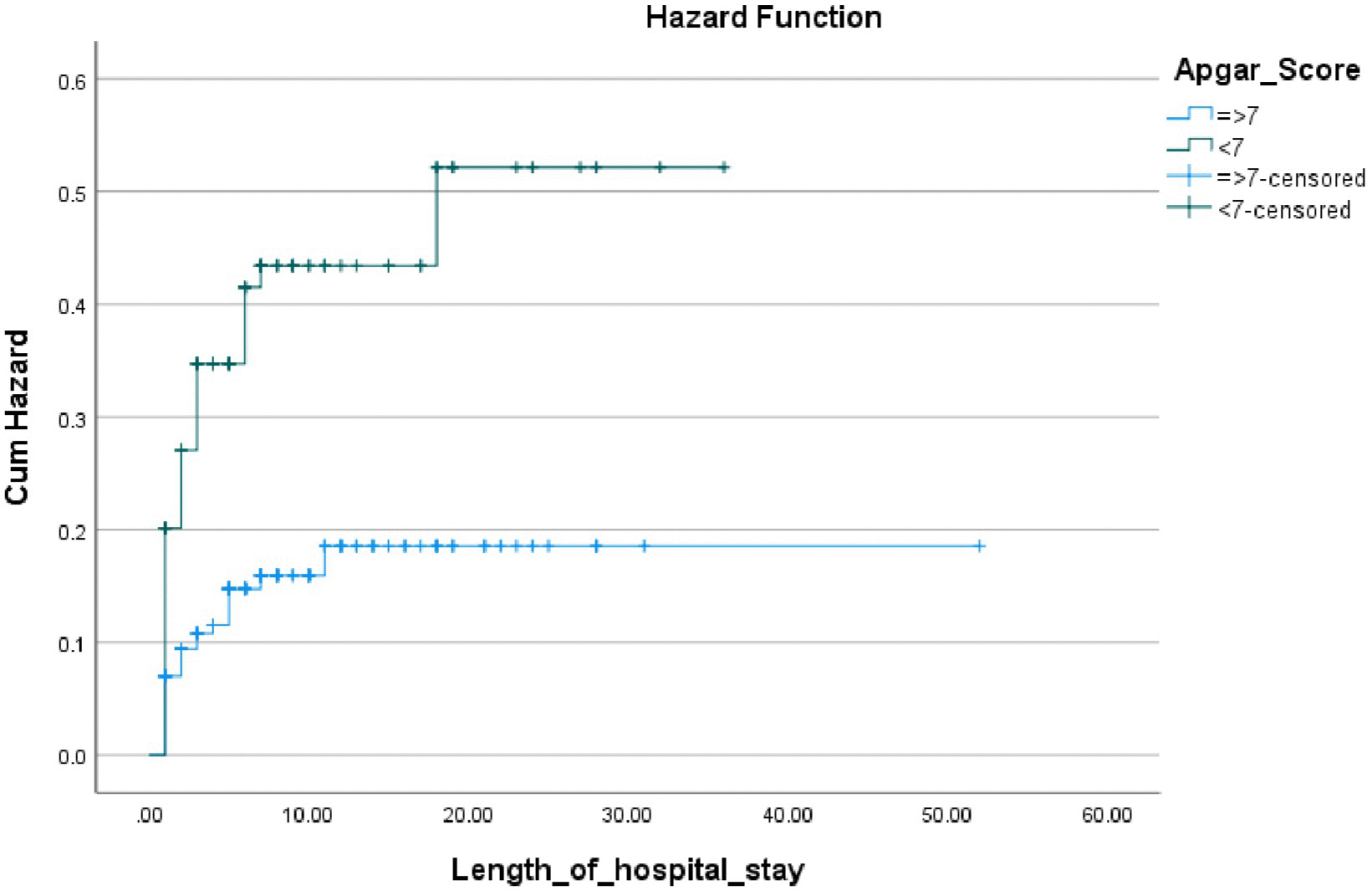
Kaplan–Meier survival curves comparing time-to-event outcomes by 5-minute APGAR score categories among low-birth-weight neonates admitted to Suhul Hospital, Shire-Inda Selassie, Northern Ethiopia, 2023.

Five-minute APGAR score and median survival time: The difference in hazard probabilities was statistically significant (p < 0.05), as validated by the log-rank test. Neonatal survival was associated with the 5-minute APGAR score, as shown in Fig 3, which presents the Kaplan–Meier survival curves stratified by APGAR score categories. A median survival time of 23.2 days (95% CI: 19.8–25.48) was observed among neonates with an APGAR score <7, whereas a significantly longer median survival of 43.8 days (95% CI: 40.58–46.97) was observed among neonates with an APGAR score 7 and above.

**Fig 4:**
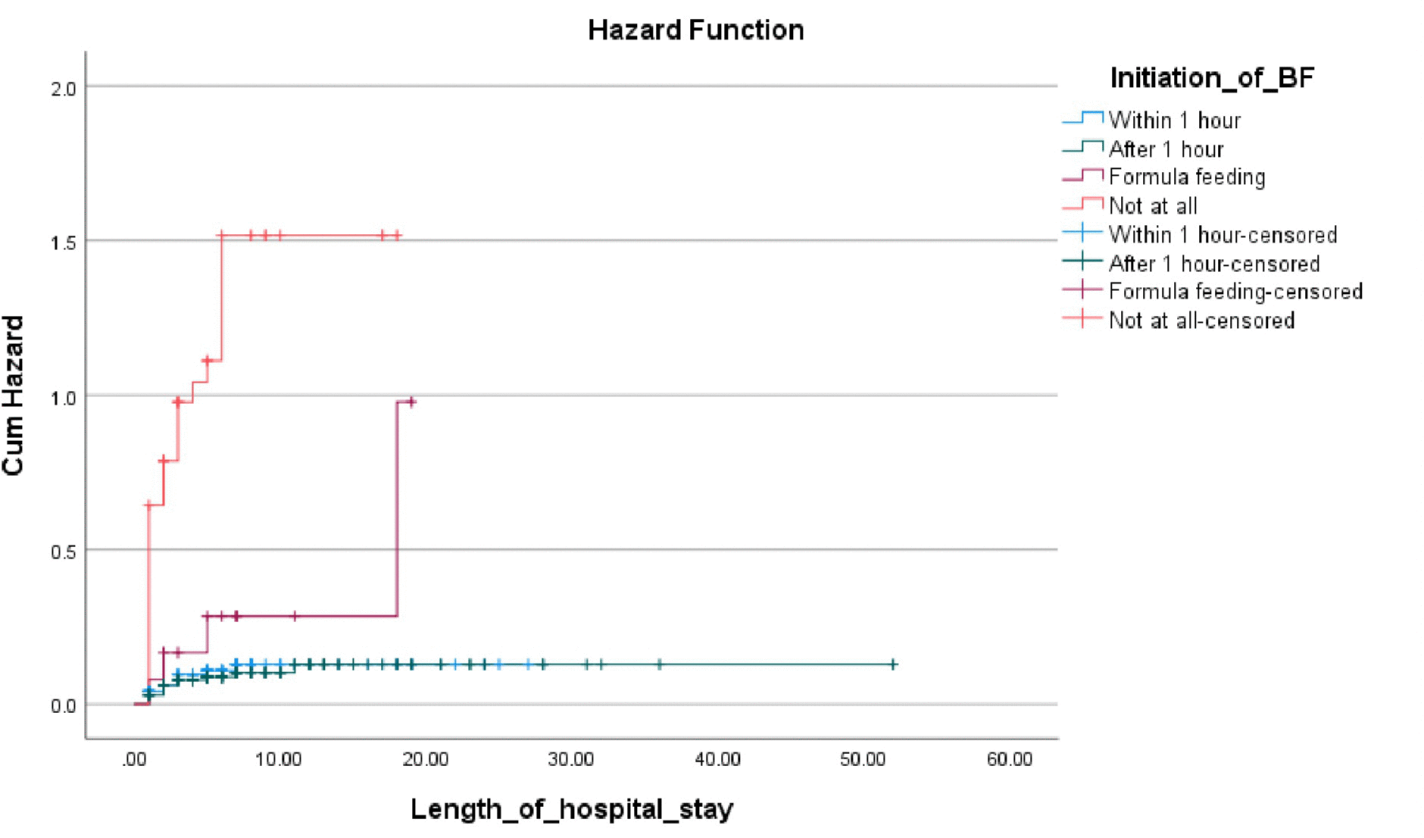
Kaplan-Meier hazard curves showing time-to-event differences stratified by breastfeeding initiation timing among low-birth-weight neonates admitted to Suhul Hospital, Shire-Inda Selassie, Northern Ethiopia, 2023.

Breastfeeding initiation and median survival time: The KM analysis showed that the timing of breastfeeding initiation had a substantial impact on newborn survival. The median survival time among newborns who were not nursed within the first hour of life was significantly lower at 5.6 days (95% CI: 3.7–7.5). On the other hand, the median survival time of neonates whose breastfeeding was initiated within the first hour of life was 24.9 days (95% CI: 23.2–26.6) (p < 0.001). Fig 4 shows the Kaplan–Meier survival curves stratified by breastfeeding initiation patterns and provides additional evidence supporting these findings.

**Fig 5:**
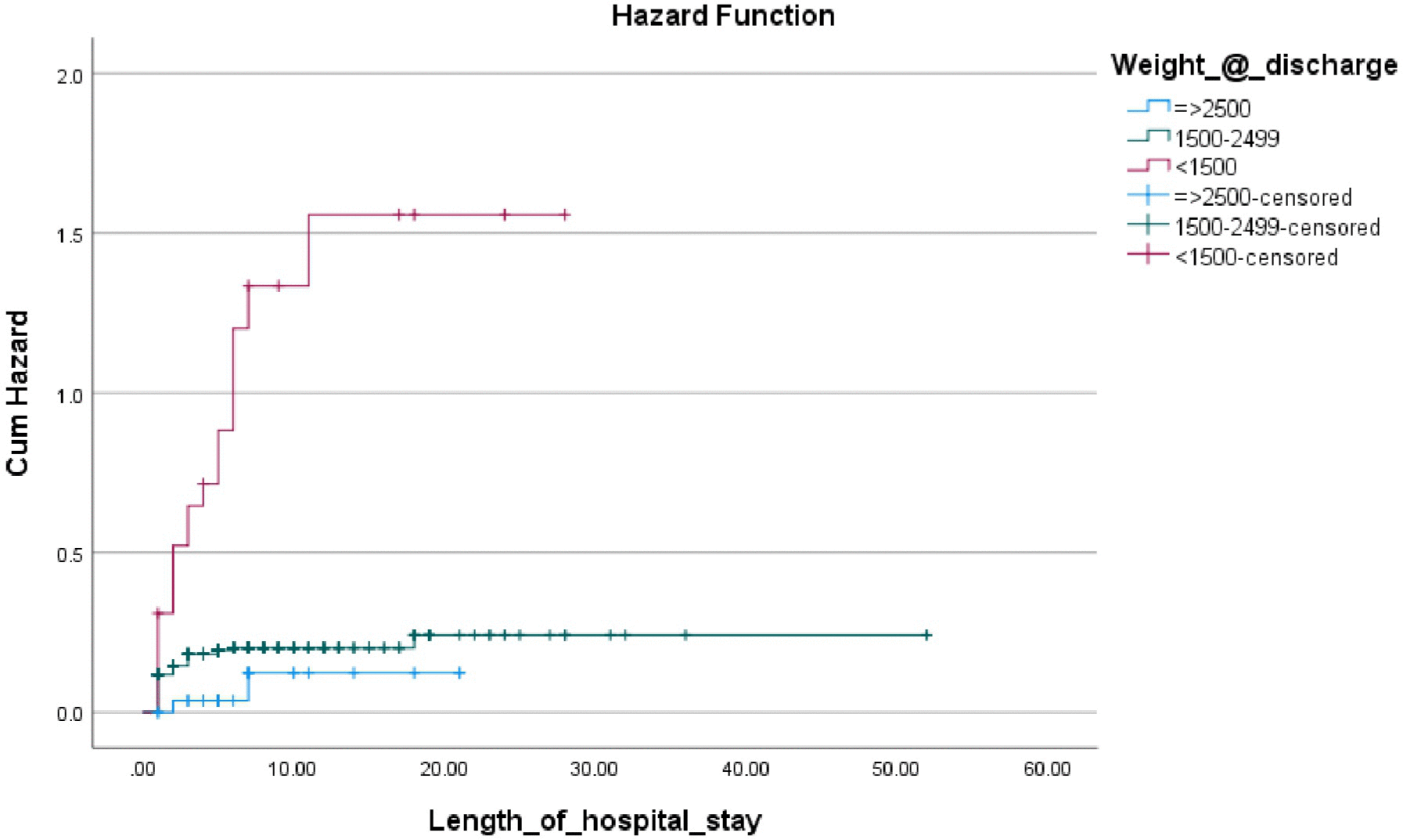
Kaplan-Meier hazard curves illustrating time-to-event differences based on weight at discharge among low-birth-weightneonates admitted to Suhul Hospital, Shire-Inda Selassie, Northern Ethiopia, 2023.

Weight at discharge and median survival time: As shown by the log-rank test group comparison, neonatal survival time differed significantly according to discharge weight. Neonates with a discharge weight of less than 1500 g experienced a significantly reduced median survival time of 8.6 days (95% CI: 4.77–12.5). Conversely, those discharged with weights ranging from 1500 to 2499 g exhibited considerably longer median survival of 41.75 days (95% CI: 38.5–44.9). These findings are visually represented in Fig 5, which illustrates the Kaplan–Meier survival curves categorized by weight at discharge.

### Evaluation of exposure-related determinants of mortality among low-birth-weight neonates

After performing multivariable Cox regression analysis, factors associated with increased neonatal mortality were identified. Initiation of breastfeeding at different levels and respiratory distress syndrome were the only significant predictors identified using the forward variable selection method. RDS was associated with an increased risk of neonatal death [AHR = 2.00, 95% CI: (1.16–3.46)]. Initiation of breastfeeding was categorized into four groups: initiation within the first hour of life (reference category), initiation after one hour [AHR = 0.744, 95% CI: (0.338–1.638)], initiation with formula feeding [AHR = 2.602, 95% CI: (0.835–8.10)], and delayed initiation of breastfeeding [AHR = 6.23, 95% CI: (3.03–12.82)] (Table 2).

**Table 2:** Cox regression analysis and mortality predictors of low-birth-weight neonates admitted to Suhul Hospital, Shire-Inda Selassie, Northern Ethiopia, 2023 [N = 329].

Variable	Category	Censored (n)	Death(n)	CHR (95% CI)	AHR (95% CI)	P value
Respiratory distress syndrome (RDS)	No	241	44	1	1	
	Yes	17	27	3.96 (3.04–7.98)	2.001 (1.16,3.46)	0.013
5-minute APGAR score	≥7	166	26	1	1	
	<7	92	45	2.58 (1.59–4.18)	1.61 (0.95–2.73)	0.073
Initiation of breastfeeding	Within 1 hour	108	12	1	1	
	After 1 hour	124	13	0.94 (0.39–1.89)	0.744 (.338,1.638)	0.46
	Formula feeding	9	4	3.08 (0.94–9.04)	2.602(0.835, 8.10)	0.056
	Delayed IBF	17	42	7.12 (5.31–19.46)	6.23(3.03, 12.82)	0.000

APGAR=Appearance, Pulse, Grimily, Activity, and Respiration, IBF=Initiation of breastfeeding, CHR= Crude hazard ratio, AHR=Adjusted hazard ratio.

## Discussion

The study assessed neonatal mortality and factors contributing to neonatal death in a humanitarian setting characterized by public health inequalities and constrained healthcare services. The main findings of this study showed a high neonatal mortality, with delayed initiation of breastfeeding and respiratory distress syndrome identified as significant predictors of mortality.

Despite incorporating multiple candidate predictors into the Cox regression model, only respiratory distress syndrome and delayed initiation of breastfeeding were retained as significant predictors using the forward variable selection method in the Cox regression model.

To the best of our knowledge, this is the first study conducted in Shire-Inda Selassie, northern Ethiopia, to assess neonatal mortality and its predictors in a conflict-affected, resource-limited setting. The neonatal mortality rate observed in this study was 216 deaths per 1,000 live births. This finding is similar to the findings reported by Mhando et al., Aguma et al., and Desalew et al. [17–19]. Moreover, the present study is comparable with the works of Eshete et al., Mehadi et al., Mengstie et al., and Mengesha et al. [20–23]. In contrast to the national neonatal mortality rate and the findings from Getahun et al., and Afzal S. and Arshad A. [5,24,25], the neonatal death in this study is substantially higher. The discrepancy in this study is most likely due to public health inequalities and fragmented health systems. The high neonatal mortality observed in this setting may also be associated with disruptions to the WHO health system building blocks, including service delivery, the health workforce, and access to medical products and technologies, resulting from conflict and resource limitations [25]. Conflict and resource limitations also affect other building blocks, including health information systems, financing, and leadership and governance. These disruptions may adversely affect maternal and newborn health outcomes by limiting access to maternal and neonatal healthcare services, essential medicines and neonatal medical supplies, health education and counseling, and quality healthcare providers [25,26]. Furthermore, the high neonatal death rate observed in this setting may also reflect factors beyond the previously discussed resource limitations and conflict-related challenges. Maternal and neonatal heterogeneity, including differences in maternal health status, healthcare-seeking behaviorrs, and neonatal clinical characteristics, may have contributed to the high neonatal mortality rate observed in this study. Differences in research methods, such as variations in study design and sample size might have an impact on the magnitude of neonatal death.

Given the high neonatal mortality observed in this study, public health inequalities and health system constraints should be addressed to reduce neonatal deaths and improve neonatal survival and health outcomes.

In the multivariable Cox regression analysis, delayed initiation of breastfeeding was found to be strongly associated with adverse neonatal outcome. The effect of delayed initiation of breastfeeding on neonatal mortality was demonstrated to be both statistically and clinically significant. In line with evidence from multiple studies, early initiation of breastfeeding provides a protective advantage for neonatal survival by improving neonatal nutritional status [27]. In contrast, delayed initiation of breastfeeding was associated with a six-fold increase in the hazard of neonatal death. The hazard ratio observed in the present study is consistent with findings reported in previous studies [18,24,28,29] despite differences in the magnitude of measures of risk and the statistical models used.

The effect size for delayed initiation of breastfeeding reported in this study was significantly greater than that reported by Mengesha et al. [29]. However, it was approximately 50% lower than that reported by Desalew et al. [19] in a similar setting.

Beyond trauma and insecurity, armed conflict disrupts the health system and limits maternal health-seeking behavior by restricting access to transportation, qualified healthcare providers, and health education and counseling on optimal breastfeeding practices, thereby adversely affecting neonatal survival [30].

Both biological and behavioral factors influence neonatal survival. Delayed initiation of breastfeeding deprives newborns of colostrum during the first hours of life, reducing passive immunity and increasing their susceptibility to infections, diseases, and death. This practice is detrimental to neonatal survival because it is inconsistent with the WHO recommendation to initiate breastfeeding within the first hour of life [30]. Early initiation of breastfeeding enables newborns to receive colostrum, which is rich in immunoglobulins, particularly immunoglobulin A (IgA) and other bioactive components that provide passive immunity, enhance neonatal immune function, and protect against infections, ultimately reducing the risk of neonatal mortality [31]. In addition, delayed initiation of breastfeeding may hinder maternal–infant bonding, thereby contributing to poorer neonatal survival outcomes [19]. Strengthening maternal health education and promoting early initiation of breastfeeding during antenatal and postnatal care are therefore essential for improving neonatal outcomes.

Maternal behavioral characteristics may also influence neonatal survival, particularly in settings with limited access to health education, awareness-raising programs, and counseling by healthcare providers on optimal breastfeeding practices [30].

As identified in the multivariable Cox proportional hazards model analysis, respiratory distress syndrome was another significant predictor of neontal mortality, with a two-fold increase in the hazard of neonatal death compared to neonates without RDS. This finding was consistent with those of several studies conducted in similar contexts [17,32].

The statistically significant association between respiratory distress syndrome and neonatal mortality observed in this study reinforces existing evidence of its clinical importance in neonatal death [33]. A plausible explanation for the increased risk is that surfactant deficiency in affected neonates leads to alveolar collapse, impaired gas exchange, and respiratory failure, thereby predisposing neonates to mortality. In resource-constrained and conflict-affected settings, shortages of essential medical supplies and neonatal care resources, including oxygen, exogenous surfactant therapy, ventilatory support, and adequately equipped neonatal intensive care units, further exacerbate this risk, contributing to poor neonatal survival outcomes [33,34].

Although predictor variables such as a 5-minute APGAR score below 7, hypothermia, birth asphyxia, and discharge weight below 1,500 grams were associated with a higher frequency of mortality in the descriptive analysis, they remained statistically insignificant in the multivariable Cox regression analysis. However, findings from previous studies have identified these independent predictors as significant contributors to neonatal mortality. In the present study, these associations were not statistically significant (p > 0.05) in the Cox regression model. Nevertheless, the clinical relevance of these predictors remains important, as they are well-recognized neonatal conditions and indicators of disease severity that may influence neonatal mortality. This lack of statistical association may be attributable to differences in study design, analytical approaches, and other methodological variations across studies. Additionally, previous studies may have been conducted in relatively stable healthcare settings that may not fully reflect the challenges of low-resource and conflict-affected environments [29,35,36].

In summary, delayed initiation of breastfeeding and respiratory distress syndrome, the significant predictors of neonatal death identified in this study, represent modifiable risk factors and critical targets for programmatic interventions aimed at reducing neonatal mortality.

**Implication of the study:** The results of this study provide valuable evidence to inform clinical and public health decision-making in conflict-affected, health system-disrupted settings. By identifying key predictors of neonatal mortality, this study highlights critical gaps in neonatal healthcare that require immediate attention from relevant stakeholders. Addressing these gaps has the potential to improve survival outcomes and strengthen neonatal care delivery within fragile health systems.

National and local government bodies could strengthen high-impact, low-cost neonatal health programs and interventions by leveraging the existing health system and locally available resources. Such efforts can be implemented through community-based channels, including health extension workers, as well as through postnatal maternal education and health promotion delivered by health providers. Moreover, this research may serve as a baseline for future studies aimed at enhancing the quality of evidence by utilizing routine primary data collected directly from newborns and their mothers.

**Limitations of the study:** This study relied entirely on secondary data derived from hospital records that were originally collected for medical purposes. Consequently, it may be subject to information bias and survivor bias. With respect to information bias, hospital records may be incomplete due to missing data or errors arising from illegible handwriting. Furthermore, in resource-limited and conflict-affected contexts such as the study setting, hospital admissions are often low, resulting in a small sample size and imprecise estimates. Regarding survivor bias, essential information on newborns may be unavailable because those who died at home from unknown causes were excluded from the analysis. These biases may have influenced the observed outcomes and the identified associated risk factors.

## Conclusion and recommendations

This study found a substantially higher incidence of neonatal death than previously reported. Multivariable Cox regression analysis identified two potentially modifiable predictors of neonatal mortality: delayed initiation of breastfeeding and respiratory distress syndrome.

Targeted public health interventions are essential to address these risk factors. Promoting the early initiation of breastfeeding through maternal education, hospital-based breastfeeding support, and community health programs may improve neonatal survival.

Likewise, strengthening the prevention, early recognition, and management of RDS by improving the capacity of health facilities, ensuring the availability of oxygen therapy, continuous positive airway pressure (CPAP), ventilator support, and surfactant therapy, as well as enhancing the skills of healthcare providers may reduce neonatal mortality in fragile health systems.

To strengthen the quality of research evidence, future studies should employ prospective cohort study designs using primary data and larger sample sizes.
